# Extensão de prazo de vigência de patentes farmacêuticas por ações judiciais: efeitos sobre compras públicas centralizadas e mercado privado

**DOI:** 10.1590/0102-311XPT231423

**Published:** 2025-01-13

**Authors:** Julia Paranhos, Lia Hasenclever, Caroline Miranda, Daniela Falcão, Lorena Abbas da Silva

**Affiliations:** 1 Instituto de Economia, Universidade Federal do Rio de Janeiro, Rio de Janeiro, Brasil.; 2 Universidade Cândido Mendes, Campos dos Goytacazes, Brasil.

**Keywords:** Patentes, Sistema Único de Saúde, Propriedade Intelectual, Patents, Unified Health System, Intellectual Property, Patentes, Sistema Único de Salud, Propiedad Intelectual

## Abstract

A inconstitucionalidade do parágrafo único do artigo 40 da Lei de Propriedade Industrial (LPI) foi declarada em 2021. Em decorrência disso, algumas empresas ajuizaram ações buscando a extensão do prazo de vigência de suas patentes farmacêuticas. O objetivo deste artigo é estimar os possíveis desdobramentos econômicos da extensão judicial do prazo das patentes nos gastos com medicamentos para as compras públicas centralizadas e o mercado privado. Esta pesquisa consiste em um estudo de caso descritivo em que foi feita uma estimativa de gastos sobre os efeitos econômicos de ações judiciais impetradas no período de 12 de maio de 2021 a 27 de julho de 2022. Como resultado, observou-se que os efeitos sobre os gastos para o Sistema Único de Saúde (SUS) e para as famílias no mercado privado são bem maiores no caso da extensão pelas demandas judiciais do que pelo extinto parágrafo único do art. 40. Considerando as três reduções de preços hipotetizadas, os gastos gerados para o SUS pelas extensões judiciais seriam 28% (redução básica), 20% (média) e 18% (drástica) maiores que pelo antigo parágrafo único do art. 40. Já para o mercado privado, os gastos decorrentes das extensões judiciais seriam 37,5% maiores na redução básica, 28,9% na média e 28,8% na drástica, que pelo parágrafo único do art. 40. Conclui-se que a extensão judicial é ainda mais prejudicial que o extinto parágrafo único, principalmente para os consumidores, nas três hipóteses de reduções de preços consideradas.

## Abstract (en)

In 2021, the sole paragraph of article 40 of the Industrial Property Law (LPI) was declared unconstitutional. As a result, some companies filed lawsuits seeking an extension of the term of validity of their pharmaceutical patents. This article aims to estimate the possible economic consequences of the extension of the term of patents in spending on medicines for centralized procurement and the private market. This research consists of a descriptive case study in which an estimate of expenses is made on the economic effects of lawsuits filed in from May 12, 2021 to July 27, 2022. As a result, it was observed that the effects on expenses for the Brazilian Unified National Health System (SUS) and for families in the private market are much greater in the case of court extension than by the extinct sole paragraph of article 40. Considering the three hypothesized price reductions, expenses generated by court extensions to the SUS account for 28% (basic reduction), 20% (average), and 18% (drastic), which are higher than the old sole paragraph of article 40 provided for. For the private market, the expenses resulting from extensions would be 37.5% higher in the basic reduction, 28.9% in the average, and 28.8% in the drastic one, when compared to the sole paragraph of article 40. It is concluded that the judicial extension is even more harmful than the extinct paragraph of article 40, especially for consumers, in the three hypotheses of price reductions considered.

## Abstract (es)

En el 2021 se declaró la inconstitucionalidad del párrafo único del artículo 40 de la Ley de Propiedad Industrial (LPI). Como resultado, algunas empresas presentaron demandas buscando extender el plazo de vigencia de sus patentes farmacéuticas. El objetivo del artículo es estimar las posibles consecuencias económicas de la extensión judicial del plazo de las patentes en los gastos en medicamentos para las compras públicas centralizadas y el mercado privado. Esta investigación consiste en un estudio de caso descriptivo en el que se realiza una estimación de gastos sobre los efectos económicos de las acciones legales interpuestas en el periodo del 12 de mayo del 2021 al 27 de julio del 2022. Como resultado, se observó que los efectos en los gastos del Sistema Único de Salud (SUS) y de las familias en el mercado privado son mucho mayores en el caso de la prórroga mediante demandas legales que bajo el extinto párrafo único del artículo 40. Considerando las tres reducciones de precios hipotéticas, los gastos generados para el SUS por las prórrogas judiciales serían un 28% (reducción básica), un 20% (media) y un 18% (drástica) superiores a los del antiguo párrafo único del artículo 40. En cuanto al mercado privado, los gastos derivados de las prórrogas judiciales serían un 37,5% superiores en la reducción básica, un 28,9% en la media y un 28,8% en la reducción drástica, que según el párrafo único del artículo 40. Se concluye que la prórroga judicial es incluso más perjudicial que el extinto párrafo único del artículo 40, especialmente para los consumidores, en las tres hipótesis de reducciones de precios consideradas.

## Introdução

Quando o Brasil implementou a Lei de Propriedade Industrial (LPI) (*Lei nº 9.279*), em 1996, para harmonizar sua legislação ao Acordo TRIPS (*Trade-Related Aspects of Intellectual Property Rights Agreement*), foram incluídos alguns dispositivos que extrapolavam o mínimo estabelecido no acordo internacional, denominados de TRIPS-plus. Entre eles, destaca-se o período mínimo de vigência de 10 anos para patentes de invenção e de sete anos para modelos de utilidade, contados da data de concessão, conforme disposto no parágrafo único do artigo 40 da LPI, doravante chamado apenas de parágrafo único do art. 40.

Em um contexto de insuficiência de examinadores do Instituto Nacional da Propriedade Industrial (INPI), essa regra fez com que diversas patentes de invenção tivessem seu prazo de vigência estendido para além dos 20 anos previstos no acordo, contados da data do depósito. Essa extensão afetou particularmente a área farmacêutica, de maneira que 92,2% das patentes farmacêuticas concedidas entre 1997 e 2018 tiveram vigência superior a 20 anos ^1^.

Em 2021, o plenário do Supremo Tribunal Federal (STF) no julgamento da *Ação Direta de Inconstitucionalidade* (ADI) *nº 5.529/DF* decidiu, por maioria de votos, pela inconstitucionalidade do parágrafo único do art. 40. No caso das tecnologias da área da saúde (produtos e processos farmacêuticos), determinou-se a retroatividade dos efeitos da decisão, isto é, a revogação das extensões já concedidas.

Como consequência, as patentes que estavam no período de extensão da vigência tiveram seu direito revogado e passaram para o domínio público. Os efeitos benéficos decorrentes dessa medida são a entrada de produtos concorrentes no mercado e a redução dos preços médios dos medicamentos ^2^, que podem levar a reduções efetivas dos custos com a aquisição de medicamentos pelo Sistema Único de Saúde (SUS) ^3^ ^,^ ^4^ ^,^ ^5^ ^,^ ^6^ e também dos gastos das famílias.

No entanto, algumas empresas farmacêuticas estrangeiras detentoras de patentes se consideraram prejudicadas pela decisão da ADI. Até 27 de julho de 2022, foram identificadas 39 ações judiciais impetradas por essas empresas, tendo como principal argumento a necessidade de reparação das possíveis perdas ocasionadas pelo atraso do INPI na análise dos pedidos de patentes. A argumentação está baseada no mecanismo para ajustamento do prazo de vigência de patentes farmacêuticas adotado pelos Estados Unidos, conhecido como *Patent Term Adjustment* (PTA), mas inexistente na legislação brasileira. Na regulamentação americana, o PTA ajusta o prazo de vigência de patentes devido ao atraso na análise do pedido por parte do escritório nacional, prorrogando em um dia para cada dia de atraso na condução do exame, e reduzindo em um dia para cada dia de atraso de resposta do requerente demandada pelo órgão examinador.

O objetivo deste artigo é estimar os possíveis desdobramentos econômicos da extensão judicial do prazo das patentes nos gastos com medicamentos para as compras públicas centralizadas e o mercado privado. A principal contribuição do artigo é a análise das consequências sobre os gastos com medicamentos nas compras governamentais e para as famílias brasileiras, auxiliando, assim, os órgãos responsáveis a tomarem decisões baseados em evidências e conscientes de suas implicações.

O artigo está estruturado em três seções, além da introdução e da conclusão. Na primeira seção, apresenta-se a metodologia do estudo de caso. Na segunda, estão seus resultados e sua análise. Na terceira, os resultados apresentados são discutidos.

## Metodologia

Esta pesquisa consiste em um estudo de caso descritivo em que se faz uma estimativa de gastos sobre os efeitos econômicos das ações judiciais impetradas por empresas farmacêuticas transnacionais entre maio de 2021 e julho de 2022 em decorrência da declaração de inconstitucionalidade, pelo STF, e consequente extinção do parágrafo único do art. 40. Este estudo se baseia nas ações judiciais para identificar as patentes e os medicamentos a serem analisados. As 39 ações são referentes a 41 patentes de 38 medicamentos de marca. Entre as 41 patentes, 39 tiveram seu período de vigência ajustado pela extinção do parágrafo único do art. 40. O objetivo é analisar os potenciais efeitos econômicos sobre os orçamentos públicos e privados da eventual concessão judicial de extensão dos prazos de vigência das patentes e discutir se os prazos de extensão solicitados estão além do que ocorreria com a vigência do parágrafo único do art. 40.

Parte-se do princípio bem fundamentado na literatura de que a extensão do prazo das patentes prolonga o monopólio dos produtos em questão, atrasa a entrada de genéricos/similares/biológicos não novos e sobrecarrega os orçamentos público e familiar ^7^ ^,^ ^8^ ^,^ ^9^. A abordagem metodológica segue a de Paranhos et al. ^6^, que mede o custo potencial desnecessário para o SUS e/ou os consumidores em continuar adquirindo os medicamentos em situação de monopólio, devido à extensão do prazo da patente, em vez de ter a possibilidade de comprar medicamentos genéricos/similares/biológicos não novos mais baratos do que seus respectivos medicamentos de marca.

As fontes de dados são: os 39 processos judiciais, o INPI, a Agência Nacional de Vigilância Sanitária (Anvisa), o Banco de Preços em Saúde (BPS) e a consultoria IQVIA Brasil (https://www.iqvia.com/pt-br/locations/brazil). As variáveis de interesse dos processos são: o número do depósito de pedido de patente, o tempo de extensão solicitado judicialmente e a data de distribuição da ação.

As ações foram levantadas pelo escritório de advogados Reis, Souza, Takeishi e Arsuffi no sistema eletrônico do Tribunal Regional Federal da 1ª Região, Seção Judiciária do Distrito Federal (PJe), por ter sido identificado que a distribuição das ações estava concentrada nesse órgão. A busca foi feita pelo nome e Cadastro Nacional da Pessoa Jurídica (CNPJ) do INPI, acessando individualmente todos os novos processos encontrados para a identificação do tema relacionado às extensões de prazo de patente devido ao atraso do INPI. O acesso foi feito às segundas-feiras e quintas-feiras entre 12 de maio de 2021 e 27 de julho de 2022, data de envio das informações para este estudo, totalizando 39 ações judiciais.

De posse dessas informações, coletou-se diretamente na base do INPI, para as 41 patentes dos processos, as datas do depósito e da concessão, além da vigência das mesmas (com base no extinto parágrafo único do art. 40 e a vigência regular de 20 anos contados a partir da data do depósito). A partir desses dados, foram calculados a diferença de vigência entre o prazo adicional solicitado judicialmente, o prazo de vigência regular da patente e o prazo de vigência baseado no extinto parágrafo único do art. 40.

Na consulta à base aberta de registros da Anvisa, verificou-se que as 41 patentes se referiam a 38 produtos, mas que apenas 35 tinham registro na agência até a data da pesquisa (setembro de 2022), dos quais: 25 são classificados como sintéticos, nove como biológicos e um como específico. Os 35 medicamentos correspondem a 33 princípios ativos (ou combinações, se for o caso), foram registrados por 17 empresas (apenas duas delas nacionais e uma importadora) e podem ser classificados em 19 distintas classes terapêuticas.

O passo seguinte foi verificar quais desses 35 medicamentos foram adquiridos nas compras públicas centralizadas e privadas entre 2017-2021. Para as compras públicas, os dados foram coletados no BPS, optando-se por selecionar apenas os princípios ativos que foram adquiridos por compra centralizada do Ministério da Saúde, executadas pelo Departamento de Logística em Saúde (DLOG). As compras públicas centralizadas englobaram apenas 24 princípios ativos ou combinações (do universo de 33 princípios ativos cobertos pelas patentes) no período, que foram agrupados de acordo com a sua apresentação comercial (concentração, substância, composição e unidade de fornecimento), e somados o valor e a quantidade totais para cada ano. Para as compras privadas, os dados foram coletados na base IQVIA Brasil, encontrando-se informações sobre as vendas no varejo para 28 dos 35 medicamentos. Esses 28 medicamentos de marca foram agrupados de acordo com a sua apresentação comercial e somados o valor e a quantidade totais para cada ano do período pesquisado. Em seguida, os valores monetários das compras públicas centralizadas e privadas foram atualizados para agosto de 2022, com base no Índice Nacional de Preços ao Consumidor (INPC) específico para produtos farmacêuticos calculado pelo Instituto Brasileiro de Geografia e Estatística (IBGE). Dessa forma, todos os valores referem-se ao ano de 2022.

Na etapa seguinte, foram calculados os gastos das compras públicas centralizadas e das vendas a varejo a partir dos indicadores de valor, quantidade e gasto médios para cada medicamento. Para estimação desses indicadores de valor, foi preciso criar indicadores de preços, já que as compras públicas centralizadas e as vendas a varejo ocorreram em vários lotes com diferentes preços e quantidades. O valor médio unitário foi estimado a partir da média dos valores unitários de cada compra ou venda, ponderada pelas respectivas quantidades e pelas diferentes concentrações, quando existentes. A quantidade média anual foi obtida a partir da divisão do total de unidades compradas pelo número de anos de incorporação ao SUS ou pelo número de anos em que houve venda no varejo, ponderando pelas diferentes concentrações. Com o valor médio unitário e a quantidade média anual, foi calculado o gasto médio anual público e privado, multiplicando esses dois valores para cada um dos medicamentos analisados.

Por fim, foram estimados os custos potenciais da extensão da vigência das patentes para o DLOG e para os consumidores, ou seja, o quanto o governo e os consumidores poderiam economizar com as reduções hipotéticas de preços, levando em conta dois cenários: o primeiro considera os prazos do extinto parágrafo único do art. 40; e o segundo os prazos adicionais solicitados nas ações judiciais. Foram, então, calculadas as reduções hipotéticas para cada princípio ativo, considerando os três níveis hipotéticos de redução do valor médio unitário: básico, médio e drástico utilizados por Paranhos et al. ^6^. Para os medicamentos sintéticos, foram considerados os seguintes percentuais para cada nível hipotético de redução: 40%, 60% e 80%, e para os biológicos: 10%, 30% e 50%. Em seguida, foi calculado o percentual das diferenças entre os custos potenciais de cada cenário.

É importante ressaltar que, diferentemente do mercado público, no mercado privado alguns medicamentos de marca já possuem sua versão genérica/similar/biológica não nova comercializada. Assim, foi possível saber a redução efetiva do valor médio unitário para esse grupo de medicamentos. Dessa forma, para esses medicamentos, foram calculadas as reduções efetivas, considerando os preços unitários das versões de genéricos, similares e biológicos não novos já comercializados no mercado brasileiro, segundo dados da IQVIA Brasil.

## Resultados e análise

As ações judiciais propostas pelas empresas farmacêuticas têm o objetivo de prolongar a vigência das patentes. Os pedidos de prazo adicional na justiça variaram de um ano, nove meses e 24 dias até 12 anos, três meses e oito dias a mais de vigência, não existindo um padrão específico para as solicitações analisadas. A média de prazo adicional solicitada pelos processos judiciais foi de sete anos.

A diferença observada entre a vigência com base no extinto parágrafo único do art. 40 e o prazo de vigência regular variou de um ano e sete meses até nove anos e oito meses a mais do que o prazo regular, sendo a média de prazo adicional de quatro anos. Assim, o tempo médio de extensão da vigência com o prazo adicional solicitado na justiça é de três anos a mais de extensão, em comparação com a vigência pelo parágrafo único do art. 40. Além disso, o prazo de vigência total da patente, somando a vigência regular de 20 anos e o período adicional solicitado judicialmente, ultrapassaria a vigência definida pelo extinto parágrafo único do art. 40 em 33 casos.

A seguir, apresenta-se a estimativa quanto aos gastos e aos custos potenciais da extensão de prazo das patentes.

### Gastos e custo potencial das extensões das patentes para as compras públicas centralizadas

Os dados analisados mostram que, entre 2017 e 2021, o gasto total do DLOG com os 24 princípios ativos analisados neste estudo foi de R$ 897,4 milhões, sendo que os gastos totais com apenas três princípios ativos (golimumabe, tofacitinibe e ustequinumabe) representam mais de 90% do gasto total. No que se refere aos valores unitários, às quantidades e aos gastos anuais médios, alguns princípios ativos se destacam. O valor médio unitário para o conjunto dos princípios ativos é de R$ 432,76, com cinco princípios ativos tendo valor médio unitário superior à média, são eles: ustequinumabe, golimumabe, lenalidomida, palbociclibe e sunitinibe. Quanto à quantidade média anual, a média geral é de 177.484, e dois princípios ativos se destacam com quantidade média anual superior à média, são eles: tofacitinibe e tenofovir alafenamida. O gasto médio anual total no período foi de R$ 251,2 milhões, mas cinco princípios ativos representam 97,8% desse valor, são eles: golimumabe, tofacitinibe, tenofovir alafenamida, etanercepte e ustequinumabe. A razão desses princípios ativos terem os maiores gasto médio anual resulta tanto da quantidade média anual demandada de alguns deles (tofacitinibe e tenofovir alafenamida) quanto do valor médio unitário de outros (golimumabe e ustequinumabe).

Na Tabela 1, são apresentados o tempo de vigência das patentes que ainda não expiraram, o tempo de extensão do extinto parágrafo único do art. 40 e do solicitado nas ações judiciais e o gasto total estimado para o DLOG com os princípios ativos analisados nos respectivos períodos de vigência da patente e de extensões.

**Tabela 1:** Gastos das compras públicas centralizadas durante a vigência regular da patente e nos cenários de extensão.

Princípio ativo	Período remanescente da vigência da patente * (anos)	Gastos na vigência das patentes (R$ milhões)	Extensão pelo parágrafo único do artigo 40 (anos)	Gastos durante a extensão pelo parágrafo único do artigo 40 (R$ milhões)	Extensão pelas ações judiciais (anos)	Gastos durante a extensão pelas ações judiciais (R$ milhões)
Apixabana	0,7	0,0016	3,2	0,0073	8,9	0,0203
Axitinibe	-	-	6,1	0,7356	11,4	1,3640
Bilastina	0,3	0,1407	5,2	2,4436	9,4	4,4342
Dapagliflozina	1,4	0,0032	4,4	0,0102	6,0	0,0141
Enzalutamida	4,2	1,9006	4,5	2,0074	10,8	4,8295
Etanercepte	3,7	58,2344	3,7	58,2344	7,9	126,4218
Golimumabe	-	-	6,6	1.012,6305	6,2	944,2266
Insulina degludeca	2,6	0,0124	5,6	0,0271	11,2	0,0542
Lenalidomida	1,3	0,1769	4,4	0,6109	5,0	0,6899
Liraglutida	2,4	0,0084	9,7	0,0336	8,8	0,0304
Macitentano	-	-	4,7	5,7355	7,4	9,0288
Metformina cloridrato associada a vildagliptina	-	-	4,3	0,0135	4,2	0,0135
Metformina cloridrato associada a dapagliflozina	1,4	0,0009	4,4	0,0029	6,0	0,0041
Mirabegrona	0,8	0,0008	5,3	0,0054	11,2	0,0115
Nintedanibe	-	-	1,7	3,7709	6,3	13,6962
Olaparibe	2,2	0,5044	4,8	1,0969	7,6	1,7341
Palbociclibe	1,0	0,3460	5,4	1,8119	9,6	3,2354
Selexipague	0,3	0,0143	4	0,1820	8,9	0,4047
Sitagliptina	2,5	0,0022	4	0,0035	5,0	0,0044
Sunitinibe	-	-	7,8	3,0791	9,2	3,6299
Tenofovir alafenamida	-	-	6,2	128,7564	5,5	113,4323
Tofacitinibe	-	-	5,7	283,3680	9,6	480,6843
Ustequinumabe	-	-	5,5	31,9328	6,2	36,3126
Vildagliptina	-	-	4,3	0,0242	4,2	0,0241
Total		61,3468		1.536,5238		1.744,3007

Fonte: elaborado pelos autores com base nos dados do Instituto Nacional de Propriedade Industrial ^15^ e Banco de Preços em Saúde ^16^.

* De 1º de janeiro de 2022 até a patente expirar.

Dos 24 princípios ativos analisados nas compras públicas centralizadas, 14 estavam, em 1º de janeiro de 2022, com patentes vigentes dentro do prazo regular, ou seja, sem qualquer extensão. Esses 14 princípios ativos representariam um gasto total de R$ 61,3 milhões para o SUS, se mantido o gasto médio anual dos anos anteriores. O princípio ativo que possui o maior prazo restante de vigência da patente é o enzalutamida (4,2 anos), seguido pelo etanercepte (3,7 anos). Esses princípios também representariam os maiores gastos totais durante essas vigências, R$ 1,9 milhão e R$ 58,2 milhões, respectivamente.

Considerando o cenário de extensão de prazo de vigência de patentes pelo extinto parágrafo único do art. 40, o princípio ativo com o maior prazo é o liraglutida (9,7 anos), seguido pelo sunitinibe (7,8 anos). Por sua vez, considerando o cenário de extensão da vigência pelas ações judiciais, o princípio ativo que possuiria maior prazo é o axitinibe (11,4 anos), seguido pelos princípios ativos insulina degludeca e mirabegrona, ambos com 11,2 anos.

No que diz respeito aos gastos totais durante as extensões pelo extinto parágrafo único do art. 40 e pelas ações judiciais, o maior gasto é com o golimumabe (R$ 1,0 bilhão pelo extinto parágrafo único do art. 40 e R$ 944,2 milhões pelas ações judiciais), seguido pelo tofacitinibe (R$ 283,4 milhões pelo extinto parágrafo único do art. 40 e R$ 480,7 milhões pelas ações judiciais).

Em relação ao custo potencial total da extensão para o SUS, considerando os valores hipotéticos de redução básica, ou seja, biológicos não novos e genéricos, respectivamente, 10% e 40% mais baratos, no primeiro cenário, de extensão pelo extinto parágrafo único do art. 40, seria possível economizar dos cofres públicos o equivalente a R$ 283,8 milhões pelo total do tempo de extensão. No segundo cenário, de extensão pelas ações judiciais, seria possível economizar dos cofres públicos o equivalente a R$ 365,6 milhões.

Considerando os valores hipotéticos de redução média, ou seja, biológicos não novos e genéricos, respectivamente, 30% e 60% mais baratos, no primeiro cenário, de extensão pelo extinto parágrafo único do art. 40, seria possível economizar dos cofres públicos o equivalente a R$ 591,1 milhões pelo total do tempo de extensão. No segundo cenário, de extensão judicial, seria possível economizar dos cofres públicos o equivalente a R$ 714,5 milhões.

Por fim, considerando os valores hipotéticos de redução drástica, ou seja, biológicos não novos e genéricos, respectivamente, 50% e 80% mais baratos, no primeiro cenário, de extensão pelo extinto parágrafo único do art. 40, seria possível economizar dos cofres públicos o equivalente a R$ 898,4 milhões pelo total do tempo de extensão. No segundo cenário, de extensão judicial, seria possível economizar dos cofres públicos o equivalente a R$ 1,1 bilhão.

### Gastos e custo potencial das extensões das patentes para o mercado privado

Os dados analisados mostram que, entre 2017 e 2021, o gasto total com os 28 medicamentos de marca que tiveram compras realizadas no mercado privado foi de quase R$ 13,4 bilhões, sendo que quatro medicamentos (Ozempic, Saxenda, Xigduo XR e Victoza) representavam mais de 45% do gasto total. No que se refere aos valores unitários, às quantidades e aos gastos anuais médios, é possível destacar alguns medicamentos de marca. O valor médio unitário do conjunto dos medicamentos analisados foi de R$ 755,09, com quatro medicamentos tendo valor médio unitário acima da média, são eles: Sylvant, Simponi, Enbrel e Revlimid. Quanto à quantidade média anual, a média geral foi de 3.510,41 e sete medicamentos se destacam com quantidade média anual superior à média geral, são eles: Xigduo XR, Forxiga, Galvus Met, Eliquis, Januvia, Alektos e Galvus. O gasto médio anual total no período foi de aproximadamente R$ 1,5 bilhão e três medicamentos (Saxenda, Ozempic e Victoza) representavam mais de 65% do gasto médio anual total.

Na Tabela 2, são apresentados o tempo de vigência das patentes que ainda não expiraram, o tempo de extensão para o extinto parágrafo único do art. 40 e para as ações judiciais e o gasto total estimado para os consumidores com os medicamentos de marca analisados em seus respectivos períodos de vigência da patente e de extensão.

**Tabela 2:** Gastos para os consumidores durante a vigência regular da patente e nos cenários de extensão.

Medicamento de referência	Período remanescente da vigência da patente * (anos)	Gastos na vigência das patentes (R$ milhões)	Extensão pelo parágrafo único do artigo 40 (anos)	Gastos durante a extensão pelo parágrafo único do artigo 40 (R$ milhões)	Extensão pelas ações judiciais (anos)	Gastos durante a extensão pelas ações judiciais (R$ milhões)
Alektos	0,3	5,09	5,2	88,45	9,4	160,51
Brintellix	0,8	11,40	4,5	67,53	4,4	66,00
Eliquis	0,7	37,83	3,2	169,07	8,9	470,98
Enbrel	3,7	17,08	3,7	17,29	7,9	36,91
Forxiga	1,4	127,04	4,4	404,25	6,0	560,00
Galvus	-	-	4,3	52,32	4,2	52,02
Galvus Met	-	-	4,3	181,06	4,2	180,02
Ibrance	1,0	23,13	5,4	121,15	9,6	216,33
Inlyta	-	-	6,1	28,80	11,4	53,40
Januvia	2,5	86,97	4,0	141,08	5,0	177,32
Lynparza	2,2	22,18	4,8	48,23	7,6	76,25
Myrbetric	0,8	10,20	5,3	65,66	11,2	138,49
Ofev	-	-	1,7	50,00	6,3	181,62
Ozempic	2,7	922,40	6,3	2.133,33	12,3	4.168,01
Qtern	1,4	11,62	4,4	36,99	6,0	51,24
Revlimid	1,3	0,18	4,4	0,62	5,0	0,70
Saxenda	2,4	960,83	9,7	3.853,11	8,8	3.483,97
Simponi	-	-	6,6	15,58	6,2	14,53
Sutent	-	-	7,8	65,92	9,2	77,72
Sylvant	0,8	0,06	8,0	0,56	7,8	0,54
Torgena	1,1	0,30	3,0	0,86	8,1	2,30
Tresiba	2,6	117,83	5,6	257,86	11,2	516,85
Uptravi	0,3	0,03	4,0	0,35	8,9	0,77
Valdoxan	3,1	34,61	5,8	64,70	5,0	55,36
Vemlidy	-	-	6,2	1,22	5,5	1,07
Victoza	2,4	595,13	9,7	2.386,56	8,8	2.157,93
Xeljanz	-	-	5,7	23,76	9,6	40,31
Xigduo XR	1,4	102,62	4,4	326,53	6,0	452,34
Total		3.086,53		10.602,88		13.393,49

Fonte: elaborado pelos autores com base nos dados do Instituto Nacional de Propriedade Industrial ^15^ e da IQVIA Brasil (https://www.iqvia.com/pt-br/locations/brazil).

* De 1º de janeiro de 2022 até a patente expirar.

Dos 28 medicamentos de marca analisados para o mercado privado, 20 estavam, em 1º de janeiro de 2022, com patentes vigentes dentro do prazo regular, ou seja, sem considerar qualquer cenário de extensão. Esses 20 medicamentos representariam um gasto total, durante a vigência das patentes, de R$ 3,09 bilhões para os consumidores, se mantido o gasto médio anual dos anos anteriores. O medicamento de marca com o maior prazo restante de vigência da patente é o Enbrel (3,7 anos), seguido pelo Valdoxan (3,1 anos). Já os medicamentos que representarão os maiores gastos totais durante as vigências das respectivas patentes são o Saxenda (R$ 960,83 milhões), seguido pelo Ozempic (R$ 922,40 milhões) e o Victoza (R$ 595,13 milhões).

Considerando o cenário de extensão de prazo de vigência de patentes pelo extinto parágrafo único do art. 40, os medicamentos de marca que teriam os maiores prazos são o Saxenda e o Victoza (9,7 anos), seguidos pelo Sylvant (8 anos). Por sua vez, considerando o cenário de extensão de prazo de vigência de patentes pelas ações judiciais, o medicamento de marca com o maior prazo é o Ozempic (12,3 anos), seguido pelo Inlyta (11,4 anos) e por Myrbetric e Tresiba, ambos com 11,2 anos.

No que diz respeito aos gastos totais durante as extensões pelo extinto parágrafo único do art. 40, o maior gasto é com o Saxenda (R$ 3,9 bilhões), seguido pelo Victoza (R$ 2,4 bilhões) e pelo Ozempic (R$ 2,1 bilhões). No que diz respeito aos gastos totais durante as extensões pelas ações judiciais, o maior gasto é com o Ozempic (R$ 4,2 bilhões), seguido pelo Saxenda (R$ 3,5 bilhões) e pelo Victoza (R$ 2,2 bilhões).

Para o mercado privado, foi possível observar a comercialização de oito medicamentos genéricos, similares e biológicos não novos concorrentes em 2022. Com isso, foram calculadas as reduções efetivas de preços e os custos potenciais da extensão das patentes dos medicamentos de marca. Destaca-se que a extensão das patentes impediria a comercialização dos medicamentos concorrentes já disponíveis no mercado. Notou-se que a redução do valor médio unitário efetiva referente aos dois medicamentos genéricos comercializados no Brasil foi de 52,72% e 76,38%; já para o biológico não novo, a redução foi de 61,49% e, para os similares, a redução variou entre -6,85% (esse valor negativo significa que o valor médio unitário do medicamento similar é superior ao valor médio unitário do respectivo medicamento de marca) e 46,91%.

No primeiro cenário de extensão do prazo de vigência de patente pelo extinto parágrafo único do art. 40, trocando os medicamentos de marca pelos seus respectivos genéricos encontrados no Brasil, seria possível gerar uma economia para os consumidores equivalente a R$ 175,77 milhões pelo total do tempo de extensão. No segundo cenário de extensão do prazo de vigência de patente pelas ações judiciais, seria possível gerar uma economia para os consumidores equivalente a R$ 444,37 milhões.

Ao trocar os medicamentos de marca pelos seus respectivos similares e biológico não novo, seria possível gerar uma economia para os consumidores equivalente a R$ 91,12 milhões pelo total do tempo de extensão no primeiro cenário. No segundo cenário, a economia para os consumidores poderia ser equivalente a R$ 250,58 milhões. O detalhamento e os demais valores são apresentados na Tabela 3.

**Tabela 3:** Custo potencial da extensão considerando preços efetivos de genéricos, similares e biológicos não novos no Brasil.

Medicamento de marca	Extensão parágrafo único do artigo 40					Extensão ações judiciais				
	Gastos (R$ milhões)	Genérico		Similar e biológico não novo		Gastos (R$ milhões)	Genérico		Similar e biológico não novo	
		Redução do valor médio unitário (%)	Custo potencial (R$ milhões)	Redução do valor médio unitário (%) *	Custo potencial (R$ milhões) **		Redução do valor médio unitário (%)	Custo potencial (R$ milhões)	Redução do valor médio unitário (%)	Custo potencial (R$ milhões)
Alektos	88,45	52,72	46,63	-3,29	-2,91	160,51	52,72	84,62	-3,29	-5,29
Brintellix	67,53	-	-	34,69	23,42	66,00	-	-	34,69	22,89
Eliquis	169,07	76,38	129,14	42,05	71,10	470,98	76,38	359,74	42,05	198,07
Enbrel ***	17,29	-	-	61,49	10,63	36,91	-	-	61,49	22,70
Forxiga	404,25	-	-	-6,85	-27,69	560,00	-	-	-6,85	-38,35
Januvia	141,08	-	-	2,65	3,73	177,32	-	-	2,65	4,69
Ofev	50,00	-	-	25,07	12,54	181,62	-	-	25,07	45,54
Revlimid	0,62	-	-	46,91	0,29	0,70	-	-	46,91	0,33
Subtotal			175,77		91,12			444,37		250,58
Total ^#^			266,89					694,94		

Fonte: elaborado pelos autores com base nos dados da IQVIA Brasil (https://www.iqvia.com/pt-br/locations/brazil).

* Os valores negativos significam que o valor médio unitário do similar é superior ao valor médio unitário do respectivo medicamento de marca;

** O custo potencial negativo sinaliza que a compra do similar representaria um custo a mais para os consumidores em relação a compra do medicamento de marca, e, por isso, está reduzindo o custo potencial total;

*** Biológico não novo;

# O total representa a soma do custo potencial para os genéricos, similares e biológicos não novos.

Analisando o conjunto dos 28 medicamentos e considerando os valores hipotéticos de redução básica, ou seja, biológicos não novos e genéricos, respectivamente, 10% e 40% mais baratos, no primeiro cenário seria possível, para os consumidores, economizar o equivalente a R$ 1,6 bilhão pelo total do tempo de extensão. No segundo cenário, a economia possível seria equivalente a R$ 2,2 bilhões também pelo total do tempo de extensão.

Considerando os valores hipotéticos de redução média, ou seja, biológicos não novos e genéricos, respectivamente, 30% e 60% mais baratos, no primeiro cenário, seria possível para os consumidores economizar aproximadamente R$ 3,8 bilhões. No segundo cenário, a economia possível seria equivalente a R$ 4,9 bilhões.

Por fim, considerando os valores hipotéticos de redução drástica, ou seja, biológicos não novos e genéricos, respectivamente, 50% e 80% mais baratos, no primeiro cenário, seria possível para os consumidores economizar aproximadamente R$ 5,9 bilhões. No segundo cenário, a economia possível seria equivalente a R$ 7,6 bilhões.

## Discussão

Os gastos com medicamentos são bastante significativos no orçamento público e nos gastos familiares. Estudos demonstram que o gasto total do Ministério da Saúde com a política de assistência farmacêutica mais do que dobrou em uma década ^10^. Em 2008, o gasto foi de R$ 9,1 bilhões e atingiu o valor de R$ 19,8 bilhões em 2019, representando 14,6% dos gastos do Ministério da Saúde nesse ano ^11^. Em relação aos gastos das famílias, de acordo com a *Pesquisa de Orçamentos Familiares* (POF) 2017-2018 ^12^, a despesa *per capita* mensal com saúde foi de R$ 133,2, sendo 35% de gastos com medicamentos e 65% com serviços de saúde. Consequentemente, torna-se ainda mais relevante considerar os efeitos de quaisquer alterações regulatórias no mercado farmacêutico a fim de verificar como e em que medida isso pode afetar o nível de preços e a concorrência, comprometendo, assim, o acesso a medicamentos e à saúde no país.

Os resultados dos potenciais prejuízos econômicos aqui apresentados são corroborados pelos achados dos estudos de Paranhos et al. ^6^, Chaves et al. ^5^ e Jannuzzi & Vasconcellos ^4^ sobre os efeitos nocivos de extensão de patentes para a saúde pública no Brasil, mas considerando outro conjunto de medicamentos. Além disso, o artigo amplia a percepção dos efeitos negativos para os consumidores que adquirem os medicamentos no mercado privado. Esse é um aspecto menos discutido na literatura, ainda que, como visto nos estudos de Vieira ^10^ e do IBGE ^12^, o peso dos medicamentos no orçamento familiar é tão relevante quanto no orçamento público. Os estudos anteriormente realizados são sobre os efeitos dos gastos públicos, mas este estudo alargou esse olhar para os efeitos sobre o mercado privado com dados secundários inéditos.

As análises dos valores, das quantidades e dos gastos médios das compras públicas centralizadas e do mercado privado reforçam a importância dos gastos com medicamentos pelas famílias no mercado privado e dos medicamentos no orçamento público. Entretanto, o mercado privado possui maior valor médio unitário e quantidade média anual, que geram maiores gasto médio anual e influenciam os custos potenciais que são mais elevados. Uma hipótese para esse resultado é que o SUS ainda não atende a determinadas demandas de necessidade de saúde, que são então atendidas pelo mercado privado. Por exemplo, os medicamentos que representaram maiores gastos no mercado privado no período (semaglutida - Ozempic, liraglutida - Saxenda, dapagliflozina+metformina - Xigduo XR e liraglutida - Victoza) não são medicamentos incorporados ao SUS, nem fazem parte da lista da Relação Nacional de Medicamentos Essenciais (Rename) 2022, apesar de ter havido compras públicas centralizadas, com quantidade média anual extremamente baixa, do liraglutida e do dapagliflozina+metformina. Apenas quatro princípios ativos possuem gasto médio anual maior no mercado público do que no privado: etanercepte, golimumabe, tenofovir alafenamida e tofacitinibe, todos devido à maior quantidade média anual nas compras públicas centralizadas. Dos quatro, os três primeiros têm parcerias de desenvolvimento produtivo (PDP) do Ministério da Saúde assinadas para transferência de tecnologia e produção local, o que pode ser a justificativa para a quantidade de compra mais alta.

Como foi observado nos resultados, os custos para o SUS e para o mercado privado são bem maiores no caso da extensão pelas ações judiciais do que no caso da extensão pelo extinto parágrafo único do art. 40. Para o SUS, as extensões pelas ações judiciais são 28,8% maiores na redução básica, 20,9% na média e 18,4% na drástica. Para o mercado privado, as extensões pelas ações judiciais são 37,5% maiores no caso da redução básica, 28,9% na média e 28,8% na drástica. Ou seja, a extensão judicial seria ainda mais prejudicial que o extinto parágrafo único do art. 40 sobretudo para os consumidores nos três cenários (Tabela 4).

**Tabela 4:** Custo potencial total da extensão da vigência das patentes para as compras públicas centralizadas e para o mercado privado.

Reduções hipotéticas	Compras públicas centralizadas			Mercado privado		
	Cenário 1: extensão pelo extinto parágrafo único do artigo 40 (R$)	Cenário 2: extensão pelas ações judiciais (R$)	Variação (%) entre cenário 2 e cenário 1	Cenário 1: extensão pelo extinto parágrafo único do artigo 40 (R$)	Cenário 2: extensão pelas ações judiciais (R$)	Variação (%) entre cenário 2 e cenário 1
Redução básica (biológicos não novos 10% e genéricos 40% mais baratos)	283,8 milhões	365,6 milhões	28,8	1,6 bilhões	2,2 bilhões	37,5
Redução média (biológicos não novos 30% e genéricos 60% mais baratos)	591,1 milhões	714,5 milhões	20,9	3,8 bilhões	4,9 bilhões	28,9
Redução drástica (biológicos não novos 50% e genéricos 80% mais baratos)	898,4 milhões	1,1 bilhão	18,4	5,9 bilhões	7,6 bilhões	28,8

Fonte: elaborado pelos autores com base nos dados do Banco de Preços em Saúde ^16^ e IQVIA Brasil (https://www.iqvia.com/pt-br/locations/brazil).

## Conclusões

Algumas questões podem ser levantadas sobre as demandas das empresas estrangeiras e os potenciais efeitos econômicos para a sociedade brasileira. A primeira é se seria válido conceder a extensão da patente baseando-se em um instituto da legislação estrangeira que não existe no ordenamento jurídico brasileiro ^13^ ^,^ ^14^. Apesar de a legislação patentária ser internacionalizada no que diz respeito aos seus parâmetros mínimos, permanece ainda a soberania nacional de regulamentação para a análise das patentes e a criação ou não de institutos tais como o PTA, que não está previsto no TRIPS. A segunda questão que cabe formular é se os pedidos de extensão estão apenas solicitando o ressarcimento do que foi perdido com o fim do parágrafo único do art. 40 ou se esse período de extensão foi definido por cada reclamante inclusive ampliando o prazo perdido. E, finalmente, caberia uma avaliação cautelosa dos efeitos econômicos para a saúde pública e privada ocasionados por uma eventual concessão dessas extensões de prazo, aspecto importante para o processo de tomada de decisão judicial devido à relevância dos gastos com medicamentos no orçamento público e das famílias brasileiras.

Esta pesquisa identificou que, para as compras públicas centralizadas, considerando as reduções hipotéticas, o cenário das extensões da vigência das patentes judicialmente representaria um gasto desnecessário para o SUS que pode variar de R$ 365,6 milhões (redução hipotética básica) até R$ 1,1 bilhão (redução hipotética drástica). Em todos os cenários estudados, os gastos seriam maiores do que na vigência do extinto parágrafo único do art. 40 da LPI, variando, respectivamente, entre 28,8%, 20,9% e 18,4%.

Para o mercado privado, o cenário é ainda mais grave com a tentativa de extensão judicial dos prazos das patentes. Considerando os preços de apenas oito medicamentos concorrentes (genéricos, similares e biológicos não novos) já disponíveis no varejo brasileiro, os custos potenciais da extensão pelas ações seriam de R$ 694,94 milhões. Se consideradas as reduções hipotéticas, as extensões judiciais dos prazos representariam um gasto desnecessário aos consumidores de R$ 2,2 bilhões (redução hipotética básica) até R$ 7,6 bilhões (redução hipotética drástica), variando, respectivamente, entre 37,5% e 28,8%.

As características observadas nos pedidos de extensão de prazo na justiça permitem concluir que a regulamentação existente antes do fim do parágrafo único do art. 40 não foi considerada para a solicitação do pedido pelos reclamantes, já que não foi possível identificar um padrão específico para as solicitações analisadas. Observou-se, também, que, em muitos casos, os prazos solicitados vão além do que seria aplicado na vigência do parágrafo único do art. 40.

O cálculo dos custos adicionais da extensão demonstra a possível economia para as compras públicas centralizadas e privadas caso as solicitações de extensão de patentes pelas vias judiciais sejam negadas. Por exemplo, foi identificada, no mercado privado brasileiro, a comercialização do similar do Revlimid em 2022, o qual tem uma redução do valor médio unitário de 46,9% em relação ao medicamento de marca, ou seja, uma economia concreta para os consumidores.

Em síntese, a análise das extensões de patente por via judicial implica em contestar a prorrogação dos prazos de vigência com base em um instituto estrangeiro e apontar as implicações do atraso na entrada de medicamentos concorrentes no mercado, desde genéricos até inovações incrementais. Como visto, as consequências disso são a ampliação, na maioria dos casos, dos prazos de vigência para além do que ocorreria com o parágrafo único do art. 40 e o aumento dos gastos com medicamentos pelo governo e famílias, reduzindo ainda mais a capacidade de compra do Ministério da Saúde e dos consumidores.

As limitações metodológicas da pesquisa são: (1) usar dados apenas das compras realizadas pelo DLOG, tendo em vista que muitos dos medicamentos analisados podem ser adquiridos por estados e municípios; e (2) considerar apenas as ações judiciais até 27 de julho de 2022. Novas pesquisas devem ser realizadas buscando avaliar como a extensão do prazo de patentes pode afetar o orçamento público e privado, assim como o acesso a medicamentos, considerando as compras estaduais e municipais e as ações judiciais impetradas após julho de 2022.

Dessa forma, recomenda-se a não concessão da extensão judicial do prazo de patentes solicitada pelas empresas com base na legislação americana, tanto pela inexistência de regulação do instituto no Brasil quanto pelos sérios prejuízos para os orçamentos do SUS e das famílias.
